# *In Vitro* Hypoglycemic and Antioxidant Activities of Dichloromethane Extract of *Xerophyta spekei*

**DOI:** 10.1155/2023/6652112

**Published:** 2023-12-29

**Authors:** Michael Musila Ndile, Wycliffe Arika Makori, Cromwell Mwiti Kibiti, Mathew Piero Ngugi

**Affiliations:** ^1^Department of Biochemistry, Microbiology and Biotechnology, Kenyatta University, P. O Box 43844-00100, Nairobi, Kenya; ^2^Department of Pure and Applied Sciences, Technical University of Mombasa, P. O. Box 90420-80100, Mombasa, Kenya

## Abstract

Diabetes mellitus is a chronic metabolic disorder which has greatly led to an increase in morbidity and mortality globally. Although *Xerophyta spekei* is widely used for the management of diabetes among the Embu and Mbeere communities in Kenya, it has never been empirically evaluated for its hypoglycemic activity. This study was carried out to verify the hypoglycemic activity of dichloromethane (DCM) extract of *Xerophyta spekei* as well as its antioxidant activity using various *in vitro* techniques. Phytochemicals associated with its antioxidant activity were identified through GC-MS. Data were subjected to descriptive statistics and expressed as mean ± standard error of the mean (*X̄* ± SEM). Comparison between various variables was performed by using unpaired Student's *t*-test and one-way analysis of variance (ANOVA), followed by Tukey's post-hoc test. The confidence interval was set at 95%. The obtained results were presented in tables and graphs. Results showed that there was no difference in *α*-amylase inhibition activity between the plant extract and the standard (IC_50_ 525.9 ± 12.34 and 475.1 ± 9.115, respectively; *p*  >  0.05). Besides, the glucose adsorption activity of the extract increased with an increase in glucose concentration (from 5.89 to 32.64 mg/dl at 5 mmol and 30 mmol of glucose, respectively; *p*  <  0.05). The extract also limited the diffusion of glucose more than the negative control (7.49 and 17.63 mg/dl, respectively; *p*  <  0.05). It also enhanced glucose uptake by yeast cells. In addition, the studied plant extract showed notable antioxidant activities. The therapeutic effects exhibited by this plant in managing diabetes mellitus and other ailments could be due to its antioxidant as well as its hypoglycemic activity. The study recommends the evaluation of *X. spekei* for *in vivo* hypoglycemic and antioxidant activities. Besides, the isolation of bioactive phytochemicals from the plant may lead to the development of new hypoglycaemic agents.

## 1. Introduction

Diabetes mellitus is a metabolic disorder characterized by high blood glucose as a result of insulin deficiency or poor insulin-directed utilization of glucose by target cells. Diabetes can be classified into type 1, type 2, gestational diabetes, and other type-specific diabetes mellitus [1]. Type 2 diabetes mellitus is the most common. It results from relative insulin resistance, inadequate insulin secretion, and excessive or inappropriate glucagon secretion. On the other hand, type 1 diabetes mellitus is characterized by absolute insulin deficiency as a result of the immune destruction of pancreatic beta cells. Diabetes mellitus is a serious problem that affects the quality of life and life expectancy. Chronic hyperglycemia leads to abnormal carbohydrate, fat, and protein metabolism, and as the disease progresses, it results in both microvascular and macrovascular complications [2]. According to the International Diabetes Federation, the prevalence of the disease among adult population was 9.3% in 2019, accounting for 463 million people worldwide. This number is expected to increase to about 700 million people by the year 2045 [3].

Several studies implicate oxidative stress as a major cause to the pathogenesis of diabetes mellitus. This is through altering enzymatic systems, causing lipid peroxidation, impairing the metabolism of glutathione, as well as decreasing vitamin C levels [4]. Complications related to increased oxidative stress include neuropathy, retinopathy, and nephropathy [5]. Besides, insulin insensitivity caused by mitochondrial dysfunction is one of the outcomes triggered by oxidative stress [6]. Metabolic abnormalities in diabetes mellitus results in an increase in superoxide anion production in the mitochondrion of endothelial cells. As a result, there is activation of polyol pathway flux, increase in advanced glycation end products (AGEs), and activation of protein kinases as well as overactivity of the hexosamine pathway [7].

With the rising prevalence of diabetes, the search for new alternative interventions is critical. In resource-limited regions, particularly in developing nations, medicinal plants play a crucial role in the treatment of diabetes. This is because they are arguably cheap, safe, and readily available [8]. On the other hand, conventional medicines used in the treatment of diabetes mellitus are marked with adverse effects including hepatotoxicity, nephrotoxicity, hypoglycemia, and gastrointestinal disturbances [9]. Furthermore, medicinal plants are rich sources of bioactive compounds with the ability to ameliorate oxidative stress resulting from diabetes. For instance, antioxidant properties associated with medicinal plants have been shown to decrease expressions of intracellular cell adhesion molecule-1 protein involved in inflammatory reactions, as well as improve diabetes state [10].

Excess production of ROS in pancreatic beta cells causes the activation of inflammatory and apoptotic transcription factors resulting in their death. Furthermore, proinflammatory cytokines produced as a result of ROS causes inflammation, atherosclerosis, vascular dysfunction, and diabetes-related kidney diseases [11]. However, medicinal plants can decrease proinflammatory cytokines and slow down the development of kidney diseases [12].

The search for new hypoglycemic agents is ever on, with medicinal plants providing new leads in finding previously unearthed phytochemicals with hypoglycemic and antioxidant effects. Currently, more than 410 medicinal plants have been reported to have antidiabetic properties [13].

Oxidative stress is a state of imbalance between free radicals and antioxidants. It results from an increase in oxidative radicals, which include reactive oxygen species and reactive nitrogen species, with the subsequent weakening of the natural antioxidant system [14]. Many studies have demonstrated that oxidative stress plays a significant role in the development of several degenerative illnesses including diabetes mellitus [15].

Both reactive oxygen species (ROS) and reactive nitrogen species (RNS) cause oxidative damage to proteins, lipids, and DNA through nitrosylation, peroxidation, carboxylation, and nitration [16]. Besides, they alter the protein structure through amino acid oxidation, free radical-induced breakage, and cross-linking. Also, peroxyl radicals and Fenton-generated OH radicals oxidize purines, pyrimidines, and deoxyribose moieties, causing the biomolecules to degrade [17].

Antioxidant enzymes such as superoxide dismutase, glutathione peroxidase, glutathione reductase, and catalase play a crucial role in humans in response to oxidative stress [16]. Besides, substances such as ascorbic acid, alpha-tocopherol, beta-carotene, and uric acid are involved in the scavenging of oxidative radicals [18]. Also, metal-binding proteins, such as albumin, ceruloplasmin, lactoferrin, and ferritin, can sequester free ions of copper, zinc, and magnesium involved in redox processes in the mitochondrial matrix [19].

Among the synthetic antioxidants available on the market are propyl gallate, tert-butylated hydroxytoluene, butyl-hydroxyquinone, butylated hydroxyanisole, and erythorbic acid. Nevertheless, they are associated with side effects such as aberrant cell division, allergies, gastroenteritis, and liver damage [14, 20].

Medicinal plants have antioxidant properties, which work in synergy with the endogenous antioxidants to bring about a redox balance in disease states [21]. Some of the mechanisms involved in stabilizing reduced radicals entails halting the initiated chain reactions by using chain-breaking antioxidants, disintegrating the oxidants into harmless products, and stabilizing transition metals involved in oxidative processes. Enzymatic antioxidants reduce the rate of chain reaction initiation by oxidants by scavenging the initial free radicals [15].

This current study aimed to evaluate the *in vitro* antioxidant and antidiabetic activities of the DCM extract of *Xerophyta spekei*. The plant *Xerophyta spekei* belongs to the family of Velloziaceae and is common in Kenya, Tanzania, Zambia, Zimbabwe, and Ethiopia [22]. The shrub measures 2–5 m in height by 6–12 cm in thickness, and its leaves are congregated at one edge. The plant is also well adapted to dry climates. It is used in South Africa by herbalists to manage pain and inflammation [22]. Among the Mbeere and Embu communities, it is used to treat wounds, snake bites, and diabetes mellitus [23]. In the Kamba community, the plant is used to treat burns [24]. Previous studies have shown that *X. spekei* has antibacterial activities against *S. aureus* and *B. subtilis* [22].

Despite its extensive use, the ability of *Xerophyta spekei* to neutralize free radicals and its hypoglycemic potential is not well known. Besides, identifying its chemical constituents is crucial not only for the development of new medicinal products but also for the discovery of valuable phytocompounds with economic benefits as well as the validation of the plant's traditional use. Therefore, the purpose of this study is to determine the antioxidant activity, *in vitro* hypoglycemic activity, and phytochemical profiles of dichloromethanolic (DCM) extract of *X. spekei*, as a potential antioxidant and hypoglycemic agent.

## 2. Materials and Methods

### 2.1. Collection of Plant Materials

Before the collection of the plant material, permission was sought from the National Commission for Science Technology and Innovation. The license number issued was NACOSTI/P/21/9972. *Xerophyta spekei* leaves and stems were collected through the help of a practicing traditional herbalist in Nthawa location, Mbeere North Sub-County in Embu County, Kenya. The global positioning system coordinates (GPS) of the collected *X*. *spekei* samples were 0^o^33′10.9″S and 37^o^37′28.5″E. Information including the local name of the plant, harvesting season, traditional uses, and preparation methods was also obtained. Plant samples were sorted, cleaned, and transported to Kenyatta University's Biochemistry, Microbiology, and Biotechnology department laboratories. The plant was identified by a qualified taxonomist at the university, and a voucher specimen was deposited at the National Museum of Kenya with voucher number: NMM/XS/001/2023.

### 2.2. Sample Preparation and Extraction

After air drying the plant material under shade for three weeks, the plant was ground into a fine powder using an electric mill. Five hundred grams of the ground powder of *X. spekei* were soaked in 1.5 L of dichloromethane with regular eddying for 24 hours. The extract was then decanted and filtered by using Whatman's filter paper no. 1 into a clean conical flask. A rotary evaporator at 40°C was used to concentrate the filtrate, and the weight of the resultant semisolid residue was assessed by using a weight balance. The extract was then stored at −20°C until the bioassay.

### 2.3. *In Vitro* Antidiabetes Determination

#### 2.3.1. Determination of *In Vitro α*-Amylase Inhibition

The *α*-amylase inhibitory activity was performed as described by Wickramaratne et al. [25] with slight modifications. Extract concentrations ranging from 0 to 1000 *μ*g/ml were made by dissolving the plant extract in 0.02M Na_2_HPO_4_/NaH_2_PO_4_ and NaCl (0.006 M) buffer at pH 6.9. A volume of 200 *μ*l of the plant extract was mixed with an equal volume of *α*-amylase solution (2 units/ml) and incubated at 30°C for 10 minutes. This was followed by the addition of 1% starch solution to each tube and incubated for 3 minutes. To terminate the reaction, 200 *μ*l of DNSA reagent (12 g of sodium potassium tartrate tetrahydrate in 8.0 mL of 2M NaOH and 20 mL of 96 mM of 3,5-dinitrosalicylic acid solution) was added, and the mixture was boiled at 85–90°C. After cooling, 5 ml of distilled water was added and the absorbance was read at 540 nm using a UV-Visible spectrophotometer. The blank was prepared at each concentration with the absence of enzyme solution. A positive control was also prepared using acarbose at similar concentrations as the extract. The % *α*-amylase inhibition was plotted against extract concentration and the IC_50_ values were calculated.

(1) $$\begin{array}{c} \%\;\alpha -\text{amylase inhibition}=\frac{\text{Absorbance of control}-\text{Absorbance of sample}}{\text{Absorbance of control}}\times 100. \end{array}$$

#### 2.3.2. Determination of *In Vitro* Glucose Adsorption Capacity

Glucose adsorption capacity was determined as described by Harish et al. [8]. In brief, *X. spekei*'s sample extract (250 mg) was added to each of the 25 ml glucose solution of increasing concentrations (5, 10, 15, 20, and 30 mmol), prepared in 50 ml conical flasks. The mixture of each concentration was stirred and incubated in a shaker water bath at 37°C for 6 h. This was followed by centrifugation for 20 minutes at 4000 × *g* and the glucose concentration in the supernatant determined. Glucose-bound was determined using the formula described by Harish et al. [8].

(2) $$\begin{array}{c} \text{Glucose bound}=\frac{G1-G6}{\text{Weight of the sample}}\times \text{Volume of the solution}, \end{array}$$

where *G*1 is the original glucose concentration and *G*6 is the glucose concentration after 6 hours

#### 2.3.3. Determination of *In Vitro* Glucose Diffusion

Glucose diffusion was carried out as described by Bhinge et al. [26]. A volume of 25 ml of glucose solution (20 mM) and plant extract samples (1%) were dialyzed in a dialysis membrane against 200 ml of distilled water in a beaker at 37°C using a shaker water bath. Contents of glucose in the dialysate were determined at intervals of 30, 60, 120, 180, and 240 minutes, using a glucose oxidase peroxidase kit. A control test was carried out without the extract.

#### 2.3.4. Determination of Glucose Uptake by Yeast Cells

A 10% (v/v) suspension of commercial baker's yeast was made by repeatedly washing commercial baker's yeast cells in distilled water through centrifugation (3000 r/m, 5 minutes) until the supernatant was clear. Various extract concentrations (1–5 mg) were added to 1 mL of glucose solution (5 and 10 mmol/L) and incubated for 10 minutes at 37°C. A volume of 100 *μ*L yeast suspension was added to the reaction, and the mixture vortexed and further incubated at 37°C for 60 min. The tubes were centrifuged (3,000 r/min, 5 min), and the glucose in the supernatant was determined as described by Cirillo [27]. The positive control was composed of metronidazole at similar concentration to the extract.

Glucose uptake by yeast cells was calculated using the following formula:

(3) $$\begin{array}{c} \text{Glucose uptake}\left(\%\right)=\frac{\text{Absorbance of control}-\text{Absorbance of sample}}{\text{Absorbance of control}}\times 100. \end{array}$$

### 2.4. *In Vitro* Antioxidant Determination

#### 2.4.1. Evaluation of DPPH Free Radical Scavenging Activity

By using an analytical balance, 12 mg of DPPH salt was weighed and dissolved in 100 ml of analytical methanol to make a concentration of 0.3 mM of DPPH solution. Of this solution, 1 ml was added to 2.5 ml of each plant extract concentration (0.01, 0.1, 1, 10, 100, and 1000 *μ*g/ml) and mixed. This was followed by incubation for 15 minutes at room temperature in a dark room. Finally, absorbance was read at 517 nm wavelength by using a Shimadzu UV-VIS (1600) microprocessor double-beam spectrophotometer. Ascorbic acid of similar concentrations as the extract was used as a positive control. To make a negative control, 2.5 ml of DPPH solution was added to 1 ml of methanol followed by reading of the absorbance. All the tests were performed in triplicates, and the percentage of radical scavenging activity (RAS) of the extract calculated as described by Kibiti and Afolayan [28].

(4) $$\begin{array}{c} \%\text{ of radical scavenging activity}=\frac{\text{Absorbance of control of the sample}}{\text{The absorbance of the control}}\times 100. \end{array}$$

#### 2.4.2. Evaluation of Ferric-Reducing Power

The ferric-reducing activity of the plant extract was determined by using the method described by Moriasi et al. [21]. The standard and extract were prepared at various concentrations (0.01, 0.1, 1, 10, 100, and 1000 *μ*g/mL). To each 1 ml of concentration of either the standard or the extract, 2.5 ml of 200 mM phosphate buffer (pH 6.6) and 2.5 ml of 30 mM potassium ferricyanide were added. The mixture was incubated at 50°C for 20 minutes. In addition, 2.5 ml of 600 mM trichloroacetic acid was added and stirred. This was followed by a 15-minute centrifugation of the mixture at 3000 rpm. 2.5 ml of the supernatant was diluted with an equal volume of distilled water. Finally, 0.5 ml of 600 mM ferric chloride was added, and the absorbance values of both the standard (ascorbic acid) and the extract measured using a spectrophotometer at 700 nm against the blank (Shimadzu UV-Vis 1600). The blank solution included all of the reagents without the extract and the standard. All the tests were performed in triplicates and ascorbic acid was used as the standard.

#### 2.4.3. Evaluation of Hydroxyl Radical Scavenging Activity

The test method was carried out as described by Arika et al. [14]. A reaction mixture comprising 100 *μ*l of 2-deoxy-2-ribose (28 mM), 20 mM KH_2_PO4-KOH buffer (pH 7.4), 200 *μ*M FeCl_3_ (1 : 1 v/v), 1.04 mM 200 *μ*l EDTA, 100 *μ*l of 1.0 mM hydrogen peroxide, 100 *μ*l of ascorbic acid (1.0 mM), and the extract of concentrate 0.1–1000 *μ*g/ml to make a total volume of 1 ml, was incubated at 37°C for 1 hour. A volume of 1.0 ml of 1% thiobarbituric acid (TBA) and 1.0 ml of 2.8% trichloroacetic acid (TCA) was added and incubated for 20 minutes at 100°C, resulting in the formation of a pink color. After cooling the solution, the optical density was measured at 532 nm. Gallic acid was employed as a positive control and was processed in the same way as the extract. The blank solution had all of the reactants but not the extract. All studies were carried out in triplicate, and the % hydroxyl radical scavenging activity was calculated as follows:

(5) $$\begin{array}{c} \%\text{ radical scavenging activity}=\frac{\text{The absorbance of control}-\text{Absorbance of sample}}{\text{absorbance of control}}\times 100. \end{array}$$

#### 2.4.4. Percentage Evaluation of Antilipid Peroxidation Activity

A volume of 2 ml of trichloroacetic acid, thiobarbituric acid, and hydrochloric acid combination (15% (w/v) of TCA, 0.375% (w/v) of TBA, and 0.25 N of HCl) was added to 1 mL of various concentrations of the standard and extract (200, 400, 600, and 800 *μ*g/ml). The mixtures were incubated in a 90°C water bath for 15 minutes before being centrifuged at 10,000 revolutions per minute for 5 minutes. Finally, using a UV-Vis spectrophotometer (Shimadzu UV-Vis 1600), the absorbance of the various supernatants was determined at 532 nm against the blank (the blank contained all the reactants apart from the extract and the standard). All the tests were performed in triplicates and the antilipid peroxidation was determined using the formula described by Moriasi et al. [21].

(6) $$\begin{array}{c} \%\text{ anti}‐\text{lipid peroxidation}=\frac{\text{Absorbance of control}-\text{Absorbance of the sample}}{\text{The absorbance of the control}}\times 100. \end{array}$$

#### 2.4.5. Evaluation of Iron Chelating Power

A volume of 1 ml of the sample extract at different concentrations (50, 100, 150, 200, and 250 *μ*g/ml) was mixed with an equal volume of 0.125 mM iron (II) sulfate. To begin the reaction, 1 ml of 0.3125 mM ferrozine was added and vortexed. This was followed by incubation at room temperature for 10 minutes. The absorbance was measured at 562 nm. EDTA was used as the positive control. The blank was composed of the reagents without the plant extract and EDTA. The tests were performed in triplicates, and the percentage of iron chelating power was determined as described by Ebrahimzadeh et al. [29].

(7) $$\begin{array}{c} \%\text{ iron }\left(\text{II}\right)\text{ chelating power}=\frac{\text{Blank absorbance}-\text{Sample absorbance}}{\text{Blank absorbance}}\times 100. \end{array}$$

#### 2.4.6. Evaluation of Hydrogen Peroxide Radical Scavenging Activity

Phosphate buffer was used to make a 40 mmol of hydrogen peroxide solution (pH 7.4.). One milliliter of various plant extract concentrations ranging from 100 to 500 ng/mL was added to 0.6 mL of hydrogen peroxide solution and incubated for 10 minutes at room temperature. The absorbance of the solution at 230 nm was measured in comparison to a blank consisting of phosphate buffer (pH 7.4). The conventional antioxidant (ascorbic acid) was treated in a similar manner. All experiments were performed in triplicates, and the hydrogen peroxide scavenging activity was estimated using the formula described by Ebrahimzadeh et al. [29].

(8) $$\begin{array}{c} \%\;H_{2}O_{2}\text{radical scavenging activity}=\frac{\text{Control absorbance}-\text{Sample absorbance}}{\text{Control absorbance}}\times 100. \end{array}$$

#### 2.4.7. Evaluation of Total Antioxidant Capacity

The phosphomolybdenum technique was employed to assess the total antioxidant capacity [30]. A solution of 0.6 M sulfuric acid, 28 mM sodium phosphate, and 4 mM ammonium molybdate (3 mL) was mixed with 0.3 mL of the extract solution. This was followed by a 90-minute incubation at 95°C. The absorbance of the solution was measured at 695 nm after cooling using a Shimadzu UV-VIS (1600) microprocessor double-beam spectrophotometer. Methanol was used in the blank solution (0.3 mL). The total antioxidant activity was calculated as the number of grams of ascorbic acid equivalent.

### 2.5. Gas Chromatography-Mass Spectrometry Analysis of the DCM Extract of *X. spekei*

The GC-MS analysis of *X. spekei* was performed using a procedure previously described by Gitahi et al. [31]. The sample was analyzed using a GC-MS (7890/5975, Agilent Technologies, Inc., Beijing, China) system, which consists of a gas chromatograph connected to a mass spectrometer. The GC-MS was outfitted with a 30 m long, 0.25 mm diameter, and 0.25 *μ*m film thickness HP-5 MS (5% phenyl methyl siloxane) low-bleed capillary column. An electron ionization system with an ionization energy of 70 Ev was used for GC-MS detection. In the split mode, the carrier gas was helium (99.99%) at a constant flow rate of 1.25 ml/min. The injector and mass transfer line temperatures were set to 250°C and 200°C, respectively, with a 1 *μ*l injection volume. With a run time of 70 minutes, the oven temperature was programmed to start at 35°C for 5 minutes and increase by 10°C/minute to 280°C for 10.5 minutes and then by 50°C/minute to 285°C for 29.9 minutes. The ion source temperature was 230°C, solvent cut time was 3.3 minutes, scan speed was 1666 Hz, scan range was 40–550 m/z, and interface temperature was 250°C. The central database of the National Institute of Standards and Technology was used to interpret the GC-MS results.

### 2.6. Data Processing and Statistical Analysis

The obtained data were entered into a Microsoft Excel spreadsheet, organized, and exported to GraphPad Prism statistical software version 8.0.2. Data were subjected to descriptive statistics and expressed as mean ± standard error of the mean (*X̄* ± SEM) after it was found to conform to basic assumptions of parametric data. For inferential statistics, one-way analysis of variance (ANOVA) followed by Tukey's post-hoc test for pairwise separation of means was performed. The confidence interval was set at 95%. Differences in antioxidant activities between the extract and the standard at different concentrations were deduced using the unpaired Student's *t*-test. The obtained results are presented in tables and graphs.

## 3. Results

### 3.1. Effect of the DCM Extract of *X. spekei* on *α*-Amylase Enzyme Activity

**Figure 1:**
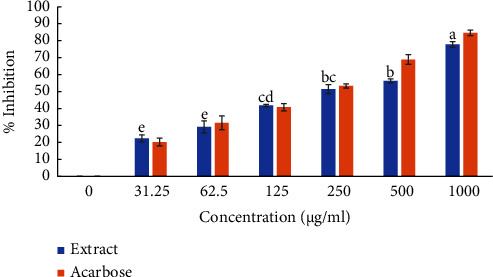
*α*-amylase enzyme % inhibition activity by the DCM extract of *X. spekei*. Bar graphs which do not share a letter across the tested concentration are statistically different (*p*  <  0.05). Bar graphs within the same concentration are not significantly different from each other (*p*  >  0.05).

The DCM extract of *X. spekei* demonstrated concentration-dependent *α*-amylase inhibition activity as shown in Figure 1. In addition, there was no significant difference in *α*-amylase inhibitory activity between extract concentrations of 37.5 *μ*g/ml and 75 *μ*g/ml, 125 *μ*g/ml, and 250 *μ*g/ml as well as between 250 *μ*g/ml and 500 *μ*g/ml (*p*  >  0.05). Besides, an extract concentration of 1000 µg/ml demonstrated the highest inhibitory activity than the rest (*p*  <  0.05). Also, there was no notable difference between the extract and the standard at similar concentrations (*p*  >  0.05). Moreover, there was no significant difference between the IC_50_ of the extract and the standard (acarbose) (*p*  >  0.05; Table 1).

### 3.2. *In Vitro* Glucose Adsorption Activity of DCM Extracts of *X. spekei*

**Figure 2:**
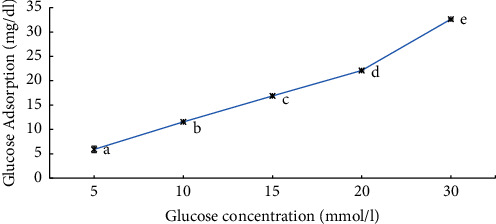
Glucose binding capacity of the DCM extract of *X. spekei* at different concentrations of glucose. Means which do not share a letter across the tested concentration are statistically different (*p*  <  0.05). Data are presented as mean ± standard error of the mean (*n* = 3).

The findings of this study indicated that the *X. spekei* extract was effective in binding glucose at both low and high concentrations. As demonstrated in Figure 2, the extract adsorbed glucose in a concentration-dependent manner. However, the adsorption capacity varied significantly between glucose concentrations (5–30 mmol/L) (*p*  <  0.05).

### 3.3. Effects of the *X. spekei's* Extract on Glucose Diffusion

**Figure 3:**
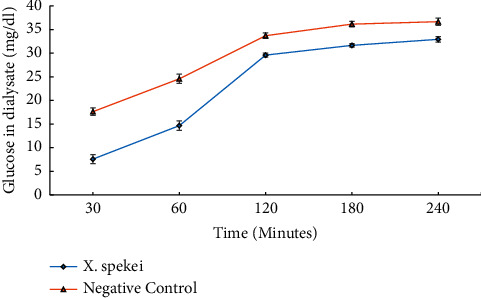
Effect of the DCM extract of *X. spekei* on glucose diffusion through dialysis membrane. Data are presented as mean ± standard error of the mean (*n* = 3).

As shown in Figure 3, the rate of diffusion of glucose across the dialysis membrane was found to be time-dependent. Besides, the inhibitory activity of the extract on glucose diffusion was significantly high compared to the control (*p*  <  0.05; Figure 3).

### 3.4. Effects of the DCM Extract of *X. spekei* on % Glucose Uptake by Yeast Cells

As indicated by the result in Table 2, glucose uptake by yeast cells at glucose concentrations of 5 mmol and 10 mmol differed significantly in all tested extract concentrations (*p*  <  0.05; Table 2). Besides, the percentage of glucose uptake by the yeast cells in both glucose concentrations (5 mmol and 10 mmol) increased remarkably with an increase in extract concentration (*p*  <  0.05; Table 2). However, it was noted that the percentage of increase in glucose uptake by yeast cells was inversely proportional to the molar concentration of glucose.

### 3.5. *In Vitro* DPPH Radical Scavenging Activity of the DCM Extract of *Xerophyta spekei*

**Figure 4:**
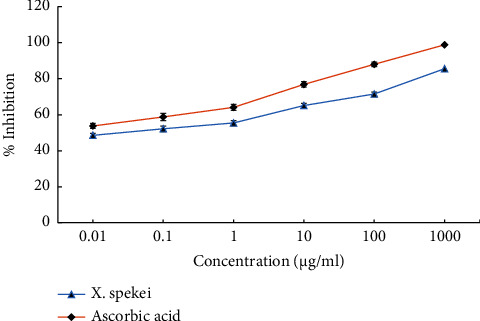
2,2-diphenyl-1-picrylhydrazyl (DPPH) radical scavenging activity of the DCM extract of *X. spekei* and standard antioxidant compound, ascorbic acid. Results are expressed as mean ± standard error of the mean (*n* = 3).

The six tested concentrations of the DCM extract of *X. spekei* displayed concentration-dependent *in vitro* DPPH radical scavenging activities as shown in Figure 4. Moreover, in all concentrations examined, the standard (ascorbic acid) demonstrated greater DPPH radical scavenging activity than the *X. spekei* extract (*p*  <  0.05). Besides, the capacity of the extract to scavenge DPPH radicals at different concentrations varied significantly (*p*  <  0.05), with the greatest concentration being the most efficient. The concentrations of the DCM extract of *X. spekei* and the standard (ascorbic acid) necessary to inhibit 50% of DPPH radicals (IC_50_) were also measured. The IC_50_ of the standard was found to be substantially greater than that of the extract (*p*  <  0.05; Table 1).

### 3.6. *In Vitro *Ferric-Reducing Activity of the DCM Extract of *Xerophyta spekei*

**Figure 5:**
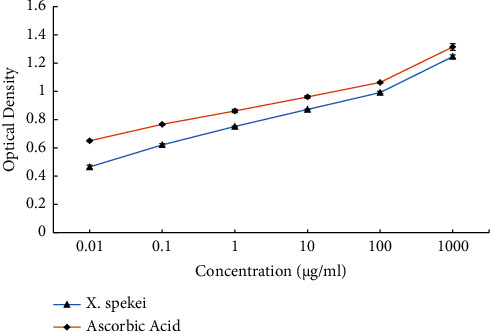
Ferric-reducing activity of the DCM extract of *X. spekei* and standard antioxidant compound, ascorbic acid. Data are presented as mean ± standard error of the mean (*n* = 3).

In Figure 5, the efficiency of ferric-reducing activity of *X. spekei* extract was demonstrated by a rise in absorbance with increasing extract concentration. Moreover, the ferric-reducing activity of the extract changed considerably across all concentrations examined (*p*  <  0.05). Nevertheless, in all concentrations examined, there was a significant difference in ferric-reducing activity between the standard (ascorbic acid) and the plant extract (*p*  <  0.05). The half-maximal effective concentration (EC_50_) of the plant extract and standard was also determined in this investigation. The EC_50_ of the standard and the extract was found to be significantly different (*p*  <  0.05; Table 1).

### 3.7. *In Vitro* Hydroxyl Radical Scavenging Activity of the DCM Extract of *Xerophyta spekei*

**Figure 6:**
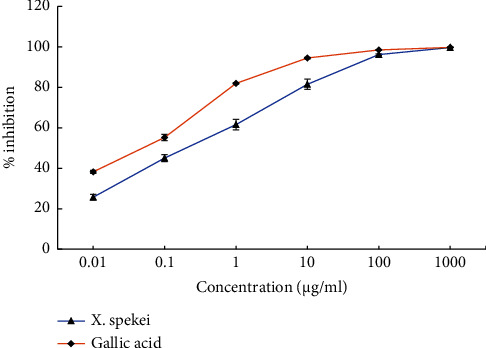
Hydroxyl radical scavenging activity of the DCM extract of *X. spekei* and standard antioxidant compound, gallic acid. Data are presented as mean ± standard error of the mean (*n* = 3).

The hydroxyl radical scavenging ability of the *X. spekei* extract was concentration-dependent, as shown in Figure 6. In addition, there was a significant difference in hydroxyl radical scavenging activity between the plant extract and the standard (gallic acid) in all tested concentrations (*p*  <  0.05), except at 1000 *μ*g/ml (*p*  >  0.05). Similarly, hydrogen radical scavenging activity across plant extract concentrations differed considerably (*p*  <  0.05), except at 100 *μ*g/ml and 1000 *μ*g/ml (*p*  >  0.05). The IC_50_ of the extract was similar to that of the standard (gallic acid) (*p*  >  0.05; Table 1).

### 3.8. *In Vitro* Antilipid Peroxidation of the DCM Extract of *Xerophyta spekei*

**Figure 7:**
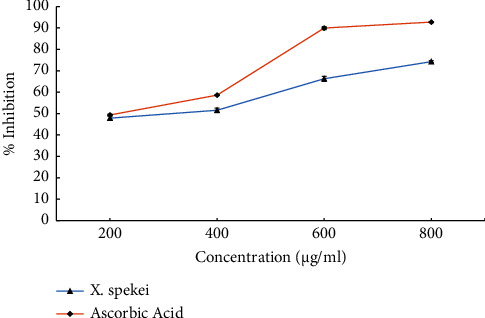
Antilipid peroxidation activity of the DCM extract of *X. spekei* and standard antioxidant compound, ascorbic acid. Data are presented as mean ± standard error of the mean (*n* = 3).

As demonstrated in Figure 7, the DCM extract of *X. spekei* inhibited lipid peroxidation in a concentration-dependent manner. All extract concentrations tested demonstrated different antilipid peroxidation activities (*p*  <  0.05). However, the antilipid peroxidation activity of the DCM extract of *X. spekei* and L-ascorbic acid at comparable concentrations revealed that the standard had significantly higher lipid peroxidation inhibition activity than the extract at all tested concentrations (*p*  <  0.05), except at 200 *μ*g/ml (*p*  >  0.05). In addition, the extract concentration necessary to inhibit 50% of lipid peroxidation activity (IC_50_) was significantly higher than that of the standard (*p*  <  0.05; Table 1).

### 3.9. *In Vitro* Iron Chelating Activity of the DCM Extract of *Xerophyta spekei*

**Figure 8:**
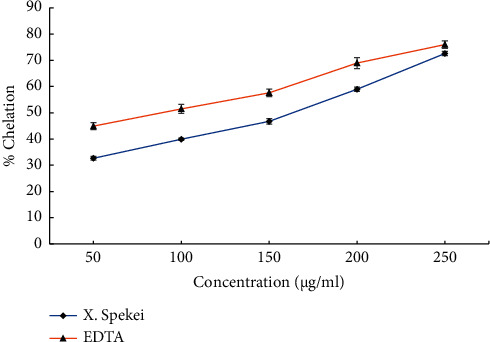
Iron chelation activity of the DCM extract of *X. spekei* and standard antioxidant compound, EDTA. Data are presented as mean ± standard error of the mean (*n* = 3).

As indicated in Figure 8, the DCM extract of *X. spekei* became more efficient in chelating iron as the extract concentrations increased. Moreover, the chelating activity varied significantly across all extract concentrations (*p*  <  0.05). All of the tested extract concentrations were considerably less efficient in iron chelating than the standard (*p*  <  0.05), except at 250 *μ*g/ml (*p*  >  0.05). The capacity of the extract and the standard to chelate 50% of radicals (IC_50_) was also assessed. Notably, the IC_50_ of the plant extract was markedly lower than that of the standard (EDTA) (*p*  <  0.05; Table 1).

### 3.10. *In Vitro* Hydrogen Peroxide Scavenging Activity of the DCM Extract of *Xerophyta spekei*

**Figure 9:**
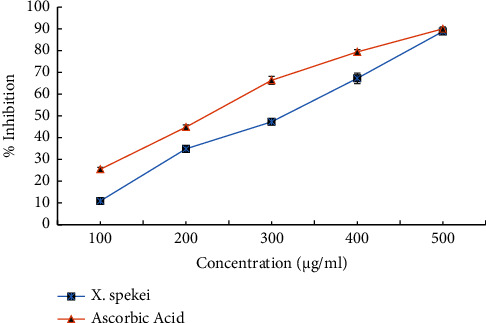
Hydrogen peroxide scavenging activity of the DCM extract of *X. spekei* and standard antioxidant compound, ascorbic acid. Data are presented as mean ± standard error of the mean (*n* = 3).

In all concentrations examined, the DCM extract of *X. spekei* was efficient in scavenging hydrogen peroxide radicals in a concentration-dependent manner. The capacity of the standard (ascorbic acid) to scavenge these radicals was considerably higher than that of the extract (*p*  <  0.05; Figure 9) at all tested concentrations, except at 500 *μ*g/ml (*p*  >  0.05; Figure 9). Also, the effectiveness of extract concentrations in scavenging hydrogen peroxide radicals differed significantly (*p*  <  0.05; Figure 9). However, the IC_50_ of the DCM extract of *X. spekei* was significantly higher than that of the standard (*p*  <  0.05; Table 1).

### 3.11. *In Vitro* Total Antioxidant Capacity of the DCM Extract of *Xerophyta spekei*

**Figure 10:**
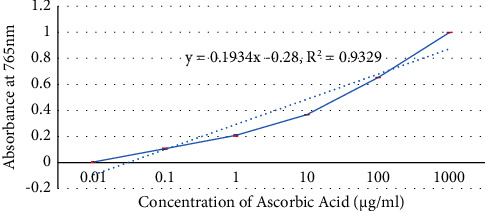
Total antioxidant capacity of the DCM extract of *X. spekei* expressed as ascorbic acid equivalence (*μ*g·AAE/ml).

In Figure 10, it was observed that the total antioxidant capacity of the extract increased with an increase in extract concentration. Besides, the total antioxidant capacity of the extract calculated from the curve equation (*y* = 0.1934x–0.2872; *R*^2^ = 0.9329) at 1000 *μ*g/ml was found to be 15.75 ± 0.035 *μ*g/mg.

### 3.12. Identification of Phytochemical Compounds in the DCM Extract of *X. spekei*

**Figure 11:**
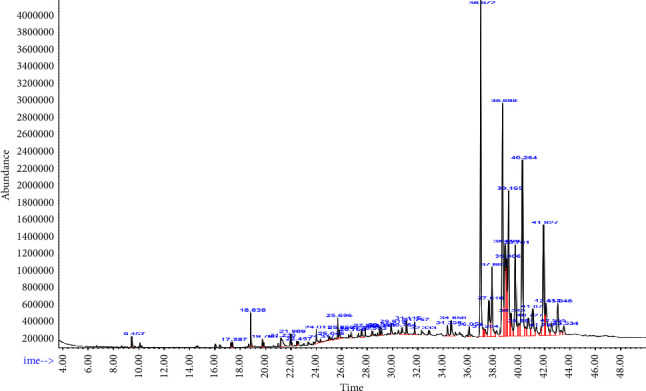
Chromatograms obtained from the gas chromatography-mass spectrometry (GC-MS) analysis of the DCM extract of *X. spekei*.

The GC-MS analysis of the DCM extract of *X. spekei* revealed several phytochemicals associated with antioxidant activity which included phenol 2,4-bis(1,1-dimethylethyl), E-15-heptadecenal, phytols, methyl stearate, dodecanoic acid, undecyl ester, coumaran-6-ol-3-one, 2-(4-hydroxy-3-methoxybenzylidene), 1H-benzoimidazole, 2-(2,4-dichlorophenoxymethyl), tetracosane, squalene, ursa-9(11),12-dien-3-one, stigmasterol, purin-2,6-dione, and 1,3-dimethyl-8-(2-nitrophenethenyl) (Table 3). Besides, Figure 11 shows the chromatograms of the identified compounds using GC-MS.

## 4. Discussion

The inhibition of carbohydrate-hydrolyzing enzymes including *α*-amylase, *α*-glycosidase, and sucrose delays carbohydrate digestion, thereby lessening postprandial blood glucose increase [46]. Acarbose, a widely used hypoglycemic agent inhibits the hydrolysis of 1,4-glycosidic linkages of starch and oligosaccharides [47].

From the study, it was found that the IC_50_ value of the extract in inhibiting* α*-amylase was similar to that of acarbose. Inhibition of the alpha-amylase activity by the plant extract could have been caused by the presence of an inhibitor in the extract fibers or encapsulation of the enzyme and starch by the extract [46]. Similar findings were reported in a study on *in vitro* hypoglycemic effects of ripe and unripe *M. sapientum* [26].

Furthermore, the finding from this study indicates that the extract can delay carbohydrate breakdown by inhibiting the *α*-amylase enzyme. Alpha-amylase inhibition activity is associated with bioactive compounds such as flavonoid and alkaloid [48]. Several studies have documented a positive correlation between phytochemicals and alpha-amylase inhibition activity [2].

Glucose diffusion retardation predicts the effect of fibers on the delay of glucose absorption in the gut [26]. In this study, the delay in glucose diffusion was greater than that of the negative control. Studies have shown that soluble dietary fibers form gel-like substances when in solution, which traps glucose molecules and prevent them from being absorbed too quickly [49]. Similarly, glucose adsorption capacity by *X. spekei* may be attributed to both the insoluble and soluble fiber contents of the extract. Previous studies have demonstrated the ability of the extracts to adsorb and retard glucose diffusion in a similar manner to that of *X. spekei*. A study on *in vitro* antidiabetic effects and antioxidant potential of *Cassia nemophila* pods showed a concentration-dependent activity on glucose adsorption [50].

Although glucose transport across the yeast cells is a facilitated process by membrane carriers [8], in this study, it was found that an increase in extract concentration resulted in an increase in glucose uptake by yeast cells. Some studies have also reported similar results. The extracts of *Cassia nemophila* pods were shown to increase glucose uptake by yeast cells in a concentration-dependent manner [50]. Furthermore, the *in vitro* hypoglycemic effects of *Albizia lebbeck* and *Mucuna pruriens* yielded comparable results [46]. Glucose uptake by yeast cells may be due to increased facilitated transport by the extract or increased cellular glucose metabolism [8]. Although glucose transport across yeast cell membranes may differ from human transport, plant extracts have been shown to increase the expression of glucose receptors in human cells, as well as enhance insulin secretion and increase the number of glucose transporters [51, 52].

Therefore, this study suggests that the *X. spekei* extract may lower postprandial hyperglycemia probably by increasing the viscosity of glucose, slowing its diffusion, and binding to glucose molecules, thus resulting in a decrease in its concentration as well as retarding the activity of alpha-amylase enzyme [25].

This study also evaluated the antioxidant potential of *X. spekei* using various *in vitro* assays. One of the assay methods included 2,2-diphenyl-1-picrylhydrazyl (DPPH) radical scavenging activity. The steady diamagnetic radical is frequently used to assess the antioxidant capabilities of both lipophilic and hydrophilic substances due to its sensitivity [14].

The extract of *X. spekei* scavenged DPPH radicals in a concentration-dependent manner. However, in all concentrations examined, the ability of the extract to scavenge these radicals was lower than that of the standard. Also, the IC_50_ of the extract was substantially greater than that of the standard. A greater IC_50_ indicates less radical scavenging activity. However, an IC_50_ value of less than 50 *μ*g/ml indicates substantial antioxidant activity [20]. The plant extract showed an IC_50_ value of 1.66 *μ*g/ml and hence demonstrated considerable activity. The presence of bioactive phytoconstituents in the plant extract was linked to its ability to scavenge DPPH radicals [53]. Similar results on DPPH radical scavenging activities were reported on *L. cornuta* aqueous root extract and methanolic extracts of *B. pinnatum* [54, 55]. A study by Arika et al. on the DCM extract of *Gnidia glauca* also revealed the ability of the extract to scavenge DPPH radicals in a concentration-dependent manner [14].

The capacity of biomolecules to contribute electrons to an oxidized substance or their oxidized intermediates determines their reducing power [14]. In the ferric-reducing experiment, the quantity of ferrous complex (Fe_4_[Fe(CN)_6_]_3_) formed, served as a demonstration of the ability of *X. spekei* extract to donate electrons. The content of the antioxidants in the extract correlated with the degree to which the complex was formed following the reduction of Fe^3+^ to Fe^2+^.

The capacity of a plant extract to transfer electrons, as indicated in this experiment, shows that it has the potential to halt oxidative chain reactions as well as the potential to decrease oxidized lipid peroxidation intermediates [14]. In parallel to this investigation, Onoja et al. established concentration-dependent ferric reduction activity in methanol leaf extract of *Bryophyllum pinnatum* [55]. Also, research on the ferric reduction activity of several chosen polyphenols yielded similar results [56].

Chelation involves combination of metal ions with organic or inorganic compounds. This allows them to be removed from intracellular and extracellular regions, enabling excretion [57]. Ion chelators scavenge reactive oxygen species and decrease accessible ions, thus reducing hydroxyl radicals produced through Fenton reactions [58]. The chelating activity of the *X. spekei* extract was concentration-dependent. Similar ion chelation activity by plant extracts was reported by Ebrahimzadeh et al. and Arika et al. [14, 29]. Furthermore, an investigation of the pulp, seeds, and fruits of *Tetrapleura tetraptera* showed that the presence of phytochemicals correlated with metal ion chelating activity [57].

Among free radicals, hydroxyl radicals are thought to be extremely reactive and capable of destroying most of the biomolecules present in the cells [59]. Hydroxyl radicals are produced via the Fenton reaction (Fe^2+^ + H_2_O_2_ ⟶ *F* e^3+^ + OH + OH•) from hydrogen peroxide or superoxide anions in the presence of metal cations. By oxidizing thiol (–SH) groups in the body, these radicals denature enzymes. Moreover, they harm cell membranes by oxidizing polyunsaturated fatty acid moieties of phospholipids [14]. They also cause lipid peroxidation, as well as protein and DNA damage.

In this study, it was found that the capacity of *X. spekei* extract to scavenge hydroxyl radicals produced through Fenton's reaction was concentration-dependent. Both the extract and the standard had equivalent hydroxyl radical scavenging activity at the highest tested concentrations. Besides, the IC_50_ of the DCM extract of *X. spekei* was equivalent to that of the standard. These findings complement those of Sasikumar who demonstrated similar effects in hydroxyl radicals scavenging activity of *Kedrostis foetidissima* leaf extracts [60]. Likewise, research studies on *G. glauca* leaf extract, diterpenoid extract of *M. glyptostroboides,* and ethanolic extract of *T. serpyllum* have also reported concentration-dependent hydroxyl radicals scavenging activity[14, 57, 61].

Lipid hydroperoxides are produced by unsaturated fatty acids, cholesterol, and esters [62]. The hydrogen atoms on methylene carbon of these intermediates actively take part in radical chain reactions that alter lipid membranes by oxidation and covalent bonding, ultimately resulting in cell death. The lipid peroxidation inhibitory activity of the DCM extract of *X. spekei* was concentration-dependent. These findings correlated with the findings of Arika *et al*, on the antilipid peroxidation activity of *G. glauca* [14]. Moreover, the outcome of this investigation was similar to the concentration-dependent lipid peroxidation inhibitory activity of unripe fruit of *R. steudneri* [63]. The antilipid peroxidation activity in this study can be ascribed to the phytochemicals present in the plant extract.

Hydrogen peroxide is a nonradical oxygen species that affects a variety of biological functions. It can permeate biological membranes, subsequently producing hydroxyl radicals in the cells. Hydrogen peroxide can be transformed into highly reactive hydroxyl radicals in the presence of transition metals such as iron. In Fenton's reaction, soluble Fe^2+^ transfers an electron to hydrogen peroxide, causing it to break down, and eventually produce hydroxyl radicals [58].

In this study, the *X. spekei* extract scavenged hydrogen peroxide in a concentration-dependent manner. A similar hydrogen peroxide scavenging ability of *Kefe cumin* extract was previously established by Ebrahimzadeh et al. [28]. Moreover, research by Arika et al. using the DCM extract of *G. glauca* produced equivalent results [14]. The presence of phytocompounds that donate electrons to hydrogen peroxide neutralizing it into water, may be the cause of the concentration-dependent hydrogen peroxide radical scavenging activity witnessed in this study.

On the other hand, some investigations have found a concentration-dependent decline in the hydrogen peroxide radical scavenging ability [28, 64]. This could be as a result of high extract concentrations saturating the reactive centers of hydroxyl radicals [64]. However, an increase in hydrogen peroxide scavenging activity of the *X. spekei* extract with an increase in extract concentration can be attributed to an increase in its active principles.

The total antioxidant capacity of an extract reflects the total amount of its bioactive constituents . At an acidic pH, the antioxidant activity of *X. spekei* extract was determined by its ability to reduce Mo (VI) to Mo (V). This is a redox reaction that occurs when an antioxidant oxidizes at the expense of an oxidant. With increasing concentration, the total *X. spekei* antioxidant capacity increased. The results are consistent with the findings of Babu *et al.*who reported on the antioxidant and free radical scavenging activities of Triphala [53].

The antioxidant potential demonstrated by the DCM extract of *X. spekei* can be ascribed to its constituent phytochemicals as revealed by GC-MS analysis. These included polyphenols, fatty acids, flavonoids, terpenes, phytosterols, and alkaloids.

Previous research has connected phenol content to antioxidant activity. According to El Jemli et al., the antioxidant activity of *J. thurifera, J. oxycedrus, J. phoenicea, and T. articulate* extracts is strongly correlated to their phenolic contents [65]. Bajpai et al. [59], reported that phenolic compounds with aromatic and hydroxyl groups are effective at scavenging hydroxyl radicals. Besides, the ability of plant extracts to effectively scavenge hydrogen peroxide is also associated with phenolic compounds, which donate electrons, thereby reducing hydrogen peroxide to water [60].

The *X. spekei* extract contained phenolic compounds including phenol, 2,4-bis(1,1-dimethylethyl), 1H-benzoimidazole, 2-(2,4-dichlorophenoxymethyl), 3-dimethylaminoanisole, and 1H-Indole, 5-methyl-2-phenyl. Derivatives of benzimidazoles are heterocyclic substances with a variety of biological functions including antioxidant, antidiabetic, antimicrobial, and anticancer activities [35–37].

Furthermore, phenol 2,4-bis(1,1-dimethylethyl) was reported to possess antioxidant activity by Alok and Suneetha [66]. Additionally, the antioxidant activity of tunichrome has been linked to phenolic compound 2,4-bis(1,1-dimethylethyl) [67]. Moreover, indole-containing phenolic compounds, such as 1H-indole and 5-methyl-2-phenyl, exhibit antioxidant properties that are enhanced by electron-donating substituents [68].

Terpenes are primarily found in essential oil hydrocarbons and are classified according to their isoprene unit (C_5_H_8_). Terpenes have been found to have a variety of health benefits in humans against diseases associated with oxidative stress [69].

Terpene compounds found in the DCM extract of *X. spekei* included phytols, squalene, ursa-9(11),12-dien-3-one, lanost-8-en-3-one, lup-20(29)-en-3-one, tirucallol, and hop-22(29)-en-3beta-ol. Previous research has shown that phytol has a high antioxidant activity, with ability to scavenge hydroxyl radicals and nitric oxide as well as inhibit the formation of thiobarbituric acid reactive substances (TBARS) [34]. Squalene, a triterpenoid with unsaturated hydrocarbon was reported to have antioxidant properties [41]. Furthermore, the cardioprotective effects of squalene are attributed to its antioxidant activity [70]. Besides, ursa-9(11),12-dien-3-one has been shown to have antioxidant activity [71].

Phytosterols are plant steroids similar to cholesterol in structure and function. They are also antioxidants and physical stabilizers of cell membranes [72]. The antioxidant activity mediated by phytosterols has been shown to protect against atherosclerosis by reducing levels of oxidized LDL-C in both *in vitro* and *in vivo* studies [44].

Additionally, the DCM extract of *X. spekei* contained stigmasterol. This is one of the most common unsaturated plant sterols with antioxidant activity and belongs to the class of tetracyclic triterpenes. Hassanein *et al.* reported that pretreating *V. faba* extracts with stigmasterol before being exposed to salt stress reduced oxidative damage and increased catalase (CAT), ascorbate peroxidase (APX), and glutathione (GSH) antioxidants [72]. Besides, the radical scavenging abilities of stigmasterol have been linked to its anticancer properties [39].

Various studies have also reported the antioxidant activities of hydrocarbons. The hydrocarbon tetracosane which was found in *X. spekei* extract was reported as having antioxidant properties [73]. In a similar study, the antioxidant activity of the alkene was also documented by Faridha Begum et al. [74].

Among compounds with antioxidant properties found in the *X. spekei* extract are fatty acids including methyl stearate, E-15-heptadecenal, dodecanoic acid, and undecyl ester. Plant-derived unsaturated fatty acids, as well as saturated fatty acids, are strongly linked to antioxidant activity [75]. Hussein and Mohammed Hamad found that extracted oil with compounds including methyl stearate and hexadecanoic acid methyl ester had significant antioxidant activity [32]. Besides its antibacterial activity, the fatty acid compound E-15-heptadecenal has also been reported to have antioxidant activity [76].

Flavonoids found in plant extracts have been linked to high DPPH radical scaveging as well as hydroxyl radical scavenging activity [77, 78]. In this study, a benzofuran flavonoid coumaran-6-ol-3-one, 2-(4-hydroxy-3-methoxybenzylidene) was identified in the DCM extract of *X. spekei*. Several biological activities, including antioxidant activity, have been associated with benzofuran derivatives [79]. In a similar study, Baliyan et al. associated the presence of flavonoids with the antioxidant activity of the extract of *Ficus religiosa* [78]. Also, Bajpai et al. linked the antioxidant activity of *Adhatoda vasica* to the flavonoids in the plant extract [59].

Alkaloids are organic compounds with nitrogen atoms. They have a variety of chemical structures as well as pharmacological activity. Antioxidant properties of alkaloids are well known. They play a role in inhibiting oxidative chain initiation, breaking chain propagation, suppressing radical formation, and protecting against oxidative stress [45, 80]. The identified alkaloids from the DCM extract of *X. spekei* included purin-2,6-dione, 1,3-dimethyl-8-[2-nitrophenethenyl], 1,1′-biphenyl,6-[(2-methylamino)ethyl] -6′-[2-phenylethyl]-2′-hydroxy-, and norpseudoephedrine. Derivatives of purine-2,6-dione have been shown to counteract TNF-*α* effects, which play a role in the production of reactive oxygen species [35]. Besides, 1,1′-biphenyl,6-[(2-methylamino)ethyl]-6′-[2-phenylethyl]-2′-hydroxy- is an indole alkaloid derivative. Indole alkaloid derivatives have demonstrated a wide range of biological activities including antioxidant activity [81].

Due to the presence of multiple phytochemicals, antioxidant activity from the plant extract is thought to be a synergistic task. The radical scavenging activity of the plant extract is also mediated through multistep processes. Plant-derived antioxidants not only combat oxidative radicals directly but also indirectly through mechanisms such as upregulating antioxidant enzymes and regulating transcription of so-called vitagenes. Furthermore, some phytochemicals have been shown to have a direct effect on the mitochondrion, an organelle susceptible to oxidative stress [82].

## 5. Conclusion

From this study, it is inferred that the DCM extract of *X. spekei* has* in vitro* hypoglycemic activity, as demostrated by its ability to inhibit* α*-amylase enzyme, prevent glucose adsorption, impede glucose diffusion, and enhance glucose transport across the cell membrane. Besides the extract was found to possess antioxidant properties which could potentially combat free radicals and restore redox homeostasis. Furthermore, the GC-MS analysis confirmed the presence of phytochemicals rich in hypoglycemic and antioxidant activities. However, there is a need to isolate bioactive compounds and determine their precise hypoglycemic and antioxidant mechanisms of action *in vivo*. Besides, isolated compounds may provide a potential lead in developing alternative therapeutic agents in future.

## Figures and Tables

**Table 1:** IC_50_ values of the DCM extract of *X. spekei* against antioxidant activity and *α*-amylase activity.

Activity	IC_50_ (*μ*g/mL)				
	Extract	Ascorbic acid	Gallic acid	EDTA	Acarbose
DPPH	1.66 ± 0.06^A^	0.98 ± 0.03^B^	—	—	—
Hydroxyl radical	0.28 ± 0.03^A^	—	0.049 ± 0.01^A^	—	—
Ferric-reducing	0.60 ± 0.002^A^	0.70 ± 0.001^B^	—	—	—
Iron chelating	70.08 ± 0.24^A^	—	—	42.54 ± 1.60^B^	—
Antilipid peroxidation	287.0 ± 8.40^A^	219.3 ± 0.77^B^	—		—
Hydrogen peroxide	248.1 ± 1.65^A^	—	—	177.0 ± 0.78^B^	—
*α*-amylase	525.9 ± 12.34^A^	—	—		475.1 ± 9.115^A^

Values are expressed as mean ± standard error of the mean (*n* = 3). Values which do not share uppercase superscript letters along the rows are significantly different (*p*  <  0.05). Data are analyzed through the unpaired *t*-test.

**Table 2:** Percentage of glucose uptake by yeast cells.

Extract concentration (mg/ml)	% glucose at 5 mM	% glucose at 10 mM
1	52.95 ± 0.74^Aa^	47.16 ± 1.37^Ab^
2	58.89 ± 1.08^Ba^	52.79 ± 2.12^Bb^
3	66.98 ± 0.50^Ca^	60.61 ± 1.00^Cb^
4	72.76 ± 1.08^Da^	66.32 ± 1.32^Db^
5	75.09 ± 0.97^Da^	68.54 ± 0.60^Db^

Values are expressed as *X̄* ± SEM. Mean values (*n* = 3) which do not share superscript small letters along the rows and superscript capital letters along the columns differ significantly from each other (*p*  <  0.05).

**Table 3:** Phytochemical compounds with antioxidant activity identified in the quantitative analysis of the dichloromethanolic (DCM) extract of *X. spekei*.

RT	Compound	Concentration (mg/g)	Reported activity
18.83	Phenol 2,4-bis(1,1-dimethylethyl)	0.30 ± 0.04	Antimicrobial, anticancer, and antioxidant [30]
24.0	E-15-heptadecenal	0.37 ± 0.05	Antioxidant [32]
25.16	Methyl stearate	0.16 ± 0.03	Antioxidant [32]
25.19	Phytols	0.90 ± 0.09	Antioxidant, antispasmodic, anticonvulsant, and anticancer [33, 34]
26.55	Dodecanoic acid and undecyl ester	1.11 ± 0.10	Antioxidant [32]
28.53	Coumaran-6-ol-3-one and 2-(4-hydroxy-3-methoxybenzylidene)	1.23 ± 0.28	Anticancer and antioxidant [30, 33]
29.62	1H-benzoimidazole, 2-(2,4-dichlorophenoxymethyl)	7.89 ± 1.57	Antioxidant, antidiabetic, antimicrobial, and anticancer [35–37]
30.77	Tetracosane	3.76 ± 0.78	Anticancer and antioxidant [38, 39]
31.12	Squalene	5.24 ± 1.06	Antioxidant, cardioprotective, anti-inflammatory, and antibacterial [40–42]
37.22	Ursa-9(11),12-dien-3-one	5.84 ± 1.18	Antioxidant and anticancer [43]
37.62	Stigmasterol	12.99 ± 2.55	Antioxidant and anticancer [39, 44]
46.6	Purin-2,6-dione, 1,3-dimethyl-8-[2-nitrophenethenyl]	6.71 ± 1.34	Antioxidant [45]

RT, retention time. Results are expressed as mean ± SEM for replicate measurement (*n* = 3).

## Data Availability

The data used to support the findings of this study are provided in this article. However, any additional information can be provided by the corresponding author upon request.

## Abbreviations

ANOVA: Analysis of variance
APX: Ascorbate peroxidase
CAT: Catalase
DCM: Dichloromethane
DPPH: 2,2-diphenyl-1-picrylhydrazyl
GDRI: Glucose diffusion retardation index
GSH: Glutathione
LDL: Low-density lipoprotein
ROS: Reactive oxygen species
SEM: Standard error of the mean
SOD: Superoxide dismutase
TBARS: Thiobarbituric acid reactive substances.

## References

[1] Baynest H. W. (2015). Classification, pathophysiology, diagnosis and management of diabetes mellitus. *Journal of Diabetes and Metabolism*.

[2] Ali H., Abu Bakar M. F., Majid M., Muhammad N., Lim S. Y. (2020). In vitro anti-diabetic activity of stingless bee honey from different botanical origins. *Food Research*.

[3] Yang L., Jiang Y., Zhang Z., Hou J., Tian S., Liu Y. (2020). The anti-diabetic activity of licorice, a widely used Chinese herb. *Journal of Ethnopharmacology*.

[4] Asmat U., Abad K., Ismail K. (2016). Diabetes mellitus and oxidative stress—a concise review. *Saudi Pharmaceutical Journal*.

[5] Johansen J. S., Harris A. K., Rychly D. J., Ergul A. (2005). Oxidative stress and the use of antioxidants in diabetes: linking basic science to clinical practice. *Cardiovascular Diabetology*.

[6] Pasupuleti V. R., Arigela C. S., Gan S. H. (2020). A review on oxidative stress, diabetic complications, and the roles of honey polyphenols. *Oxidative Medicine and Cellular Longevity*.

[7] Giacco F., Brownlee M., Schmidt A. M. (2010). Oxidative stress and diabetic complications. *Circulation Research*.

[8] Harish M., Ahmed F., Urooj A. (2014). In vitro hypoglycemic effects of Butea monosperma Lam. Leaves and bark. *Journal of Food Science and Technology*.

[9] Chaudhury A., Duvoor C., Reddy Dendi V. S. (2017). Clinical review of antidiabetic drugs: implications for type 2 diabetes mellitus management. *Frontiers in Endocrinology*.

[10] Nasri H., Shirzad H., Baradaran A., Rafieian-kopaei M. (2015). Antioxidant plants and diabetes mellitus. *Journal of Research in Medical Sciences*.

[11] Bailey C. J., Day C. (2018). Treatment of type 2 diabetes: future approaches. *British Medical Bulletin*.

[12] Putra I. M. W. A., Fakhrudin N., Nurrochmad A., Wahyuono S. (2023). A review of medicinal plants with renoprotective activity in diabetic nephropathy animal models. *The Life*.

[13] Jacob B., Narendhirakannan R. T. (2019). Role of medicinal plants in the management of diabetes mellitus: a review. *Biotech*.

[14] Arika W., Kibiti C. M., Njagi J. M., Ngugi M. P. (2019). In vitro antioxidant properties of dichloromethanolic leaf extract of *Gnidia glauca* (fresen) as a promising antiobesity drug. *Journal of Evidence-Based Integrative Medicine*.

[15] Afolabi O., Oloyede O. (2014). Antioxidant properties of the extracts of Talinum Triangulare and its effect on antioxidant enzymes in tissue homogenate of swiss albino rat. *Toxicology International*.

[16] Rains J. L., Jain S. K. (2011). Oxidative stress, insulin signaling and diabetes. *Free Radical Biology and Medicine*.

[17] Gangwar M., Gautam M. K., Sharma A. K., Tripathi Y. B., Goel R. K., Nath G. (2014). Antioxidant capacity and radical scavenging effect of polyphenol rich *Mallotus philippenensis* fruit extract on human erythrocytes: an *in vitro* study. *The Scientific World Journal*.

[18] Pisoschi A. M., Pop A., Cimpeanu C., Predoi G. (2016). Antioxidant capacity determination in plants and plant-derived products: a review. *Oxidative Medicine and Cellular Longevity*.

[19] Yeung A. W. K., Tzvetkov N. T., El-Tawil O. S., Bungǎu S. G., Abdel-Daim M. M., Atanasov A. G. (2019). Antioxidants: scientific literature landscape analysis. *Oxidative Medicine and Cellular Longevity*.

[20] Lidon F., Silva M. (2016). An overview on applications and side effects of antioxidant food additives. *Emirates Journal of Food and Agriculture*.

[21] Moriasi G., Ireri A., Ngugi M. P. (2020). In vitro antioxidant activities of the aqueous and methanolic stem bark extracts of *Piliostigma thonningii* (schum.). *Journal of Evidence-Based Integrative Medicine*.

[22] Nyalo P. O., Omwenga G. I., Ngugi M. P. (2023). Antibacterial properties and GC-MS analysis of ethyl acetate extracts of Xerophyta spekei (Baker) and Grewia tembensis (Fresen). *Heliyon*.

[23] Kareru P. G., Kenji G. M., Gachanja A. N., Keriko J. M., Mungai G., Mungai G. (2006). Traditional medicines among the Embu and Mbeere peoples of Kenya. *African Journal of Traditional, Complementary and Alternative Medicines: AJTCAM*.

[24] Kisangau D. P., Herrmann T. M. (2007). Utilization and conservation of medicinal plants used for primary health care in Makueni district, Kenya. *International Journal of Biodiversity Science and Management*.

[25] Wickramaratne M. N., Punchihewa J. C., Wickramaratne D. B. M. (2016). In-vitro alpha amylase inhibitory activity of the leaf extracts of *Adenanthera pavonina*. *BMC Complementary and Alternative Medicine*.

[26] Bhinge S. D., Bhutkar M. A., Randive D. S., Wadkar G. H., Hasabe T. S. (2018). In vitro hypoglycemic effects of unripe and ripe fruits of Musa sapientum. *Brazilian Journal of Pharmaceutical Sciences*.

[27] Cirillo V. P. (1962). Mechanism of glucose transport across the yeast cell membrane. *Journal of Bacteriology*.

[28] Kibiti C. M., Afolayan A. J. (2015). Preliminary phytochemical screening and biological activities of *Bulbine abyssinica* used in the folk medicine in the eastern cape province, South Africa. *Evidence-based Complementary and Alternative Medicine*.

[29] Ebrahimzadeh M., Nabavi S., Nabavi S., Eslami B., Rahmani Z. (2010). Antioxidant and antihaemolytic activities of the leaves of *Kefe cumin (Laser trilobum* L) umbelliferae. *Tropical Journal of Pharmaceutical Research*.

[30] Jayathilake A. L., Jayasinghe M. A., Walpita J. (2022). Development of ginger, turmeric oleoresins and pomegranate peel extracts incorporated pasteurized milk with pharmacologically important active compounds. *Applied Food Research*.

[31] Gitahi S. M., Ngugi M. P., Mburu D. N., Machocho A. K. (2021). Contact toxicity effects of selected organic leaf extracts of tithonia diversifolia (hemsl.) A. Gray and vernonia lasiopus (O. Hoffman) against sitophilus zeamais motschulsky (Coleoptera: Curculionidae). *International Journal of Zoology*.

[32] Bahreldin M. (2020). GC-MS analysis, antimicrobial and antioxidant activity of sudanes adansoina digitata L; (malvaceae) fixed oil. *Saudi Journal of Medical and Pharmaceutical Sciences*.

[33] Vasanthakumar S., Dineshkumar G., Jayaseelan K. (2019). Phytochemical screening, GC-MS analysis and antibacterial evaluation of ethanolic leaves extract of *Avicennia marina*. *Journal of Drug Delivery and Therapeutics*.

[34] Santos C. C. D. M. P., Salvadori M. S., Mota V. G. (2013). Antinociceptive and antioxidant activities of phytol in vivo and in vitro models. *Neuroscience Journal*.

[35] Chłoń-Rzepa G., Jankowska A., Ślusarczyk M. (2018). Novel butanehydrazide derivatives of purine-2,6-dione as dual PDE4/7 inhibitors with potential anti-inflammatory activity: design, synthesis and biological evaluation. *European Journal of Medicinal Chemistry*.

[36] Pathare B., Bansode T. (2021). Review- biological active benzimidazole derivatives. *Results in Chemistry*.

[37] Wójcik-Pszczoła K., Jankowska A., Ślusarczyk M. (2021). Synthesis and in vitro evaluation of anti-inflammatory, antioxidant, and anti-fibrotic effects of new 8-aminopurine-2,6-dione-based phosphodiesterase inhibitors as promising anti-asthmatic agents. *Bioorganic Chemistry*.

[38] Siddiqui M. A., Siddiqui H. H., Mishra A., Usmani A. (2020). Evaluation of cytotoxic activity of lavandula stoechas aerial parts fractions against HepG2 cell lines. *Current Bioactive Compounds*.

[39] Paudel M. R., Chand M. B., Pant B., Pant B. (2019). Assessment of antioxidant and cytotoxic activities of extracts of dendrobium crepidatum. *Biomolecules*.

[40] Srivastava A., Subhashini S S., Keshari A. K., Srivastava R. (2021). Phytochemical and GC-MS analysis of hydro ethanolic leaf extract of ocimum sanctum (L.). *Pharmacognosy Research*.

[41] Naina Mohamed I. (2021). Interdependence of anti-inflammatory and antioxidant properties of squalene–implication for cardiovascular health. *The Life*.

[42] Farvin K. H. S., Surendraraj A., Anandan R. (2009). Protective effect of squalene on endogenous antioxidant vitamins in experimentally induced myocardial infarction in rats. *Asian Journal of Biochemistry*.

[43] Musini A., Rao J. P., Giri A. (2015). Phytochemicals of Salacia oblonga responsible for free radical scavenging and antiproliferative activity against breast cancer cell lines (MDA-MB-231). *Physiology and Molecular Biology of Plants*.

[44] Ashraf R., Bhatti H. N., Mushtaq M., Anwar F. (2021). Chapter 10—stigmasterol. *A Centum of Valuable Plant Bioactives*.

[45] Yin T.-P., Cai L., Xing Y. (2016). Alkaloids with antioxidant activities from *Aconitum handelianum*. *Journal of Asian Natural Products Research*.

[46] Bhutkar M., Bhise S. (2013). In vitro hypoglycemic effects of Albizzia lebbeck and Mucuna pruriens. *Asian Pacific Journal of Tropical Biomedicine*.

[47] Ou S., Kwok K., Li Y., Fu L. (2001). In vitro study of possible role of dietary fiber in lowering postprandial serum glucose. *Journal of Agricultural and Food Chemistry*.

[48] Kulkarni A. A., Kamble R. P. (2021). *α*-Amylase inhibitory secondary metabolites from artemisia pallens wall ex DC—biochemical and docking studies. *Biology and Life Sciences Forum*.

[49] Ahmed F., Sairam S., Urooj A. (2011). In vitro hypoglycemic effects of selected dietary fiber sources. *Journal of Food Science and Technology*.

[50] Rehman G., Hamayun M., Iqbal A. (2018). In vitro antidiabetic effects and antioxidant potential of *Cassia nemophila* Pods. *BioMed Research International*.

[51] Purintrapiban J., Keawpradub N., Kansenalak S., Chittrakarn S., Janchawee B., Sawangjaroen K. (2011). Study on glucose transport in muscle cells by extracts from Mitragyna speciosa (Korth) and mitragynine. *Natural Product Research*.

[52] Ighodaro O. M. (2018). Molecular pathways associated with oxidative stress in diabetes mellitus. *Biomedicine and Pharmacotherapy*.

[53] Babu D., Gurumurthy P., Borra S. K., Cherian K. M. (2013). Antioxidant and free radical scavenging activity of triphala determined by using different in vitro models. *Journal of Medicinal Plants Research*.

[54] Akimat E. K., Omwenga G. I., Moriasi G. A., Ngugi M. P. (2021). Antioxidant, anti-inflammatory, acute oral toxicity, and qualitative phytochemistry of the aqueous root extract of launaea cornuta (hochst. Ex oliv. & hiern.). *Journal of Evidence-Based Integrative Medicine*.

[55] Onoja S. O., Ihejirika G. Q., Nwankudu O. N., Omeh Y. N., Ezeja M. I. (2018). Antidiarrheal and antioxidant activities of methanol extract of Bryophyllum pinnatum leaf harvested from south-eastern Nigeria in mice. *Journal of Pharmaceutics*.

[56] Pandey K. B., Rizvi S. I. (2012). Ferric reducing and radical scavenging activities of selected important polyphenols present in foods. *International Journal of Food Properties*.

[57] Adusei S., Otchere J. K., Oteng P., Mensah R. Q., Tei-Mensah E. (2019). Phytochemical analysis, antioxidant and metal chelating capacity of Tetrapleura tetraptera. *Heliyon*.

[58] Adjimani J. P., Asare P. (2015). Antioxidant and free radical scavenging activity of iron chelators. *Toxicology Reports*.

[59] Bajpai V. K., Sharma A., Kang S. C., Baek K.-H. (2014). Antioxidant, lipid peroxidation inhibition and free radical scavenging efficacy of a diterpenoid compound sugiol isolated from Metasequoia glyptostroboides. *Asian Pacific Journal of Tropical Medicine*.

[60] Sasikumar V. (2014). Evaluation of free radical scavenging activity of various leaf extracts from Kedrostis foetidissima (jacq.) cogn. *Biochemistry and Analytical Biochemistry*.

[61] Tanuj J., Vijay J. (2022). Evaluation of hydroxyl radical scavenging activity of ethanolic extract of thymus serpyllum. *International Journal of Pharmaceutical Sciences and Research*.

[62] El-Aal H. A. H. M. A. (2012). Lipid peroxidation end-products as a key of oxidative stress: effect of antioxidant on their production and transfer of free radicals. *Lipid Peroxidation*.

[63] Raghavendra H. L. R., Kekuda T. R. P. (2018). Antiradical and lipid peroxidation inhibitory activity of ripe and unripe fruit of rubus steudneri schweinf. (Rosaceae). *Pharmacognosy Journal*.

[64] Muthoni Guchu B., Machocho A. K., Mwihia S. K., Ngugi M. P. (2020). In vitro antioxidant activities of methanolic extracts of Caesalpinia volkensii harms., vernonia lasiopus O. Hoffm., and Acacia hockii de wild. *Evidence-based Complementary and Alternative Medicine*.

[65] El Jemli M., Kamal R., Marmouzi I., Zerrouki A., Cherrah Y., Alaoui K. (2016). Radical-scavenging activity and ferric reducing ability of juniperus thurifera (L.), J. Oxycedrus (L.), J. phoenicea (L.) and tetraclinis articulata (L.). *Advances in Pharmacological Sciences*.

[66] Alok P., Suneetha V. (2014). Punica granatum (pomegranate) rind extract as a potent substitute for LAscorbic acid with respect to the antioxidant activity. *Research Journal of Pharmaceutical, Biological and Chemical Sciences*.

[67] Marhamati Z., Marhamatizadeh M. H., Mohebbi G. (2021). Evaluation of the physicochemical, antioxidant, and antibacterial properties of tunichrome released from *Phallusia nigra* Persian gulf marine tunicate. *Journal of Food Quality*.

[68] Arora G., Sharma S., Sahni T., Sharma P. (2018). Antioxidant and antimicrobial activity of some 2-phenyl-1H-indoles and benzimidazoles. *Indian Journal of Pharmaceutical Sciences*.

[69] González-Burgos E., Gómez-Serranillos M. (2012). Terpene compounds in nature: a review of their potential antioxidant activity. *Current Medicinal Chemistry*.

[70] Yoshida Y., Niki E. (2003). Antioxidant effects of phytosterol and its components. *Journal of Nutritional Science and Vitaminology*.

[71] Berger A., Jones P. J., Abumweis S. S. (2004). Plant sterols: factors affecting their efficacy and safety as functional food ingredients. *Lipids in Health and Disease*.

[72] Hassanein R. A., Hashem H. A., Khalil R. R. (2012). Stigmasterol treatment increases salt stress tolerance of faba bean plants by enhancing antioxidant systems. *Plant Omics*.

[73] Ochieng Nyalo P., Isanda Omwenga G., Piero Ngugi M. (2022). GC-MS analysis, antibacterial and antioxidant potential of ethyl acetate leaf extract of Senna singueana (delile) grown in Kenya. *Evidence-based Complementary and Alternative Medicine*.

[74] Faridha Begum I., Mohankumar R., Jeevan M., Ramani K. (2016). GC–MS analysis of bio-active molecules derived from paracoccus pantotrophus FMR19 and the antimicrobial activity against bacterial pathogens and MDROs. *Indian Journal of Microbiology*.

[75] Henry G. E., Momin R. A., Nair M. G., Dewitt D. L. (2002). Antioxidant and cyclooxygenase activities of fatty acids found in food. *Journal of Agricultural and Food Chemistry*.

[76] Godara P., Dulara B. K., Barwer N., Chaudhary N. S. (2019). Comparative GC-MS analysis of bioactive phytochemicals from different plant parts and callus of leptadenia reticulata wight and arn. *Pharmacognosy Journal*.

[77] Kumar S., Pandey A. K. (2013). Chemistry and biological activities of flavonoids: an overview. *The Scientific World Journal*.

[78] Baliyan S., Mukherjee R., Priyadarshini A. (2022). Determination of antioxidants by DPPH radical scavenging activity and quantitative phytochemical analysis of Ficus religiosa. *Molecules*.

[79] Miao Y., Hu Y., Yang J., Liu T., Sun J., Wang X. (2019). Natural source, bioactivity and synthesis of benzofuran derivatives. *Royal Society of Chemistry Advances*.

[80] Daley S., Cordell G. A. (2021). Alkaloids in contemporary drug discovery to meet global disease needs. *Molecules*.

[81] Kumar S (2020). A brief review of the biological potential of indole derivatives. *Future Journal of Pharmaceutical Sciences*.

[82] Franco R., Navarro G., Martínez-Pinilla E. (2019). Hormetic and mitochondria-related mechanisms of antioxidant action of phytochemicals. *Antioxidants*.

